# Female Sex and IL28B, a Synergism for Spontaneous Viral Clearance in Hepatitis C Virus (HCV) Seroconverters from a Community-Based Cohort

**DOI:** 10.1371/journal.pone.0027555

**Published:** 2011-11-15

**Authors:** Charlotte H. B. S. van den Berg, Bart P. X. Grady, Janke Schinkel, Thijs van de Laar, Richard Molenkamp, Robin van Houdt, Roel A. Coutinho, Debbie van Baarle, Maria Prins

**Affiliations:** 1 Cluster Infectious Diseases, Department of Research, Center for Infection and Immunity Amsterdam (CINIMA), Public Health Service, Amsterdam, The Netherlands; 2 Department of Infectious Diseases, Tropical Medicine and AIDS, CINIMA, Academic Medical Center, Amsterdam, The Netherlands; 3 Department of Medical Microbiology, CINIMA, Academic Medical Center, Amsterdam, The Netherlands; 4 Cluster Infectious Diseases, Laboratory of Public Health, Public Health Service, Amsterdam, The Netherlands; 5 Department of Medical Microbiology and Infection Control, VU University Medical Center, Amsterdam, The Netherlands; 6 Center for Infectious Disease Control, National Institute of Public Health and the Environment, Bilthoven, The Netherlands; 7 Department of Immunology, University Medical Center Utrecht, Utrecht, The Netherlands; 8 Department of Internal Medicine, University Medical Center Utrecht, Utrecht, The Netherlands; 9 Department of Infectious Diseases, Tropical Medicine and AIDS, CINIMA, Academic Medical Center, Amsterdam, The Netherlands; Institut Pasteur, France

## Abstract

**Background & Aims:**

Since acute hepatitis C virus (HCV) infection is often asymptomatic, it is difficult to examine the rate and determinants of spontaneous clearance. Consequently, these studies are subject to bias, which can potentially lead to biased rates of viral clearance and risk estimates. We evaluated determinants of spontaneous HCV clearance among HCV seroconverters identified in a unique community-based cohort.

**Methods:**

Subjects were 106 drug users with documented dates of HCV seroconversion from the Amsterdam Cohort Study. Logistic regression was used to examine sociodemographic, behavioral, clinical, viral and host determinants, measured around acute infection, of HCV clearance.

**Results:**

The spontaneous viral clearance rate was 33.0% (95% confidence interval (CI) 24.2–42.8). In univariate analyses female sex and fever were significantly associated with spontaneous clearance. The favorable genotypes for rs12979860 (CC) and rs8099917 (TT) were associated with spontaneous clearance, although borderline significant. In multivariate analysis, females with the favorable genotype for rs12979860 (CC) had an increased odds to spontaneously clear HCV infection (adjustedOR 6.62, 95% 2.69–26.13), whereas females with the unfavorable genotype were as likely as men with the favorable and unfavorable genotype to clear HCV. Chronic Hepatitis B infection and absence of HIV coinfection around HCV seroconversion also favor HCV clearance.

**Conclusions:**

This study shows that co-infection with HIV and HBV and genetic variation in the IL28B region play an important role in spontaneous clearance of HCV. Our findings suggest a possible synergistic interaction between female sex and IL28B in spontaneous clearance of HCV.

## Introduction

Hepatitis C virus (HCV) is mainly transmitted through exposure to infected blood [1]. Acute infection is usually asymptomatic and can lead to chronic infection in an estimated 75% of individuals [2], [3]. Chronic HCV infection can in time lead to liver fibrosis and cirrhosis, end-stage liver disease, and hepatocellular carcinoma [4].

Treatment success rates are higher when individuals are treated during acute HCV than when they are treated during chronic infection [5]–[7]. To be able to decide whether early treatment is indicated, early predictors of spontaneous viral clearance are urgently needed.

The majority of the studies on spontaneous viral clearance have been conducted among anti-HCV positive individuals, for whom the exact moment of anti-HCV seroconversion is unknown. These prevalence studies are subject to selection bias, which can potentially lead to biased rates of viral clearance and risk estimates [8]. Studies among acute HCV cases are less likely to suffer from methodological flaws. However, the potential to examine the rate and determinants of spontaneous viral clearance of acute HCV infection is restricted, since acute infection is usually asymptomatic and therefore rarely recognized. The limited published data indicated that 14–42% of persons with acute HCV cleared the virus, and that clearance is associated with symptomatic acute HCV, female sex, non-black race, lower peak HCV-RNA titer, induction of neutralizing antibodies early in HCV infection, and high and broad HCV-specific CD4^+^ and CD8^+^ T-cell responses [7], [9]–[17]. Recently, several studies demonstrated that genetic variations in the region near the interleukin-28B (IL28B) are associated with HCV treatment response [18]–[21]. IL 28B gene encodes interferon (IFN)-λ3, which is related to IFN-α and IFN-β. Two single nucleotide polymorphisms (SNPs), rs12979860 and rs8099917, located upstream of the IL28B gene have also been associated with spontaneous clearance [19], [22]–[25]. Although the sample size in some of these studies is not limited, all but one [24] have been conducted among individuals with prevalent HCV infection [19], [22], [25] or in a selected subgroup [23] and lack precise longitudinal data around HCV seroconversion.

Since the prospective Amsterdam Cohort Study (ACS) among drug users (DU) has retrospectively identified a substantial number of incident HCV infections [26] it provided a unique opportunity to study the spontaneous HCV clearance rate and its potential sociodemographic, behavioral, clinical, viral and host determinants (including coinfections), measured before and around acute HCV infection, in a population that includes asymptomatic acute HCV cases.

## Methods

### Ethics statement

The medical ethics committee of the Academic Medical Center (MEC AMC) approved the current study.

### Study population

The ACS among DU is an open, prospective cohort study initiated in 1985 to investigate the prevalence, incidence, and risk factors of human immunodeficiency virus (HIV) infections and other blood-borne and/or sexually transmitted diseases, as well as the effects of interventions [27]. All ACS participants provide written informed consent. ACS participants visit the Amsterdam Health Service every 4–6 months; they complete a standardized questionnaire about their health, risk behavior, and sociodemographic situation. Questions at ACS entry refer to the 6 months preceding the visit; questions at follow-up refer to the interim since the preceding visit. Blood is drawn each visit for laboratory testing and storage.

### Screening for HCV, HBV and HIV

To identify HCV seroconverters, we retrospectively tested stored serum from all participants having at least two visits between December 1985 and November 2005 (n = 1276). Individuals who were anti-HCV negative at ACS entry were tested for antibodies at their most recent ACS visit. On finding seroconversion, we tested samples taken between these two visits to determine the moment of seroconversion (third generation commercial microparticle EIA system, AxSym HCV version 3.0; Abbott, Wiesbaden, Germany). All HCV seroconverters were included in the present study (n = 59). Also included, were DU who were anti-HCV positive at ACS entry and had started injecting drug use within 2 years before entry. Since we have shown that approximately 50% of DU acquire HCV infection within 2 years after starting injecting drug use [26], the latter group most likely represent recent HCV infections.

To assess hepatitis B status, stored blood samples were retrospectively tested for anti-HBc (AxSym Core, Abbott, Germany and Hepanostika; Organon Technika, the Netherlands) by the same algorithm as for HCV. To identify individuals with an active hepatitis B virus (HBV) infection, the presence of HBV surface antigen (HBsAg) was determined (AxSym HBsAg, Abbott, Germany) in serum. All ACS participants (n = 1,640) were prospectively tested for HIV antibodies by enzyme linked immunosorbent assays (ELISA) at each visit. Results since 1986 have been confirmed by Western blot using HIV Blot version 2.2, Genelab diagnostics (Singapore).

### Reverse-transcription polymerase chain reaction (RT-PCR) methods

For each seroconverter, HCV RNA was measured at a minimum of 4 time-points when samples were available: the last visit before HCV seroconversion, i.e., the last anti-HCV negative visit, two visits shortly after HCV seroconversion and a visit approximately 1 year after HCV seroconversion. In those who were anti-HCV positive at entry, HCV RNA was measured at, at least, 2 time-points: at study entry and the consecutive visit(s). All serum samples were tested for the presence of HCV RNA using an in-house quantitative real-time RT-PCR based on the conserved 5′-UTR, and HCV genotyping was performed as described by Van de Laar *et al.* [28] The nucleotide sequence data have been deposited in the GenBank sequence database under accession numbers JN547478-JN547481 and JN657313 t/m JN657415.

### Il28B genotyping

Single nucleotide polymorphism (SNP) genotyping was performed using Allelic Discrimination assays from Applied Biosystems for the SNPs rs8099917 and rs12979860 following the instructions of the manufacturer. Genotyping for rs8099917 was performed using predesigned assays (Applied Biosystems, assay ID “C__11710096_10”).

Genotyping for rs12979860 was performed using Taqman custom-designed primers and probes as follows: forward primer GCCTGTCGTGTACTGAACCA, reverse primer GCGCGGAGTGCAATTCAAC, and probes TGGTTC*G*CGCCTTC (VIC) and CTGGTTCACGCCTTC (FAM) (Applied Biosystems).

Before performing the PCR reactions DNA is added with Allelic Discrimination Assay Mix and TaqMan Universal PCR Master Mix to MicroAmp Optical 96-Well Reaction Plates (Applied Biosystems). Real-Time PCR reactions were performed using the ABI Prism 7900HT system (Applied Biosystems). After preheating for 10 min. at 95°C, 40 cycles of 15 seconds at 95°C and one minute at 60°C followed. Data were analyzed using the ABI PRISM SDS software v.2.2.1 (Applied Biosystems).

### Statistical analyses and definitions

Date of HCV seroconversion was estimated as the midpoint between the last anti-HCV negative visit and the first anti-HCV positive visit in HCV seroconverters. In recent HCV cases who started injection ≤2 years prior to enrolment, the date of seroconversion was estimated as the midpoint between start of injection drug use and entry in ACS. In the group of HCV seroconverters, spontaneous HCV clearance was defined as 2 consecutive HCV RNA-negative test results, at least 4 months apart, after HCV seroconversion. In the group with recent HCV infection, clearance was defined as 2 consecutive HCV RNA-negative test results after ACS entry. HCV viral persistence is defined as the continuous presence of HCV RNA at one or two of these visits.

Logistic regression was used to evaluate the associations between spontaneous clearance of HCV and sociodemographic variables at the first anti-HCV positive visit: drug use related variables, standard collected data on clinical symptoms, HCV characteristics at first visit after seroconversion or study entry, co-infections and IL28B (rs12979860 and rs8099917). Multivariate logistic regression models were built using backward stepwise techniques. All variables with a p-value≤0.20 in univariate analysis were considered for entry into the model. Statistical analysis was performed by use of STATA (version 11.1; StataCorp) and SPSS (version 17.0; SPSS Inc.) software. A p-value≤0.05 was considered to be statistically significant. Interaction and confounding were checked between the variables in the final models.

Furthermore, using Poisson regression, we examined whether the incidence of each clinical symptom reported at visits up to 2 years following HCV seroconversion was higher for DU who cleared HCV after acute infection compared with those who did not. For the HCV seroconverters, we used the last visit before HCV seroconversion and the first 3 visits following HCV seroconversion for these analyses. Since DU could contribute more visits and events, Generalized Estimating Equations (GEE) was used to correct for repeated measurements within subjects.

In a sensitivity analysis, analyses were repeated using only the HCV seroconverters who had a small seroconversion interval (i.e., no more than 6 months between last anti-HCV negative visit and first anti-HCV positive visit) (n = 36).

## Results

### General characteristics

Sufficient follow-up and serum were available to assess outcome of acute HCV infection for 55 out of 59 HCV seroconverters and for 51 of 58 recent HCV infected DU. The median interval between last negative and first positive visit was 4.0 months (interquartile range (IQR) 3.7–5.0 months) for the 55 HCV seroconverters. The median duration of injecting drug use before study entry for DU with recent HCV infection was 1.12 years (IQR 0.33–1.50 years). Of all 106 participants, 41.5% were female, and the majority was of west-European ethnicity (84.9%). The median age at HCV seroconversion was 28.5 years (IQR 24.7–34.2 years). Of 106 participants, 93 (87.7%) reported recent injecting drug use, of whom 50.5% reported daily injecting and 34.6% reported recent sharing of needles. None of the 106 participants received HCV treatment in the first two years following HCV seroconversion.

Of those that were HCV-RNA-positive around HCV seroconversion or ACS entry, 42.5% had HCV genotype 1, 35.0% had genotype 3, 7.5% had genotype 2, and 7.5% had genotype 4. For the 6 samples in which HCV genotype could not be determined, HCV viral load was <1,000 IU/ml. The median log viral load (IQR) at the first available visit after seroconversion or ACS entry did not differ significantly among genotypes (p = 0.25, Kruskal-Wallis) being 4.40 (3.38–5.42), 5.73 (4.64–6.14), 4.36 (3.19–5.30) and 4.69 (3.00–5.66) for genotypes 1, 2, 3, and 4, respectively. Median baseline alanine aminotransferase (ALT) levels for those who developed cHCV was 31.0 IU/L (IQR 17.3–89.5) and 12.0 IU/L (IQR 7.50–29.0) for those who spontaneously resolved HCV, p = 0.002 [29].

#### Rate and determinants of spontaneous viral clearance

According to our definition of at least 2 consecutive HCV-RNA negative test results shortly after HCV seroconversion, the infection was spontaneously cleared in 35 of the 106 DU (33.0%, 95% CI 24.2–42.8%).

#### Sociodemographics and behavior

In univariate analysis, women had threefold higher odds of spontaneous viral clearance (see table 1) than men. Of 38 HIV-negative women, 50.0% cleared HCV spontaneously, in contrast to only 25.5% of HIV-negative men. Having a steady partner who injected or did not inject, was also significantly associated with higher odds of HCV clearance compared to not having a steady partner (twofold and threefold higher odds, respectively).

**Table 1:** Univariate analysis of sociodemographic factors, behavioral factors and clinical symptoms associated with HCV clearance in a cohort of 106 individuals with acute HCV acquired through injection drug use.

		N	Clearance rate (%)	OR (95%CI)	P value
Age (per 10 year increase)		106	33.0	0.62 (0.32–1.18)	0.13
Sex	Male	62	22.6	1	0.007
	Female	44	47.7	3.13	
Ethnicity	Western European	90	31.1	1	0.33
	Non-Western European	16	43.8	1.72 (0.58–5.09)	
Calendar year of infection	≤1988	43	30.2	1	0.63
	1989–1991	25	44.0	1.81 (0.65–5.04)	
	1992–1994	21	28.6	0.92 (0.29–2.91)	
	≥1995	17	29.4	0.96 (0.28–3.29)	
Jaundice	No	32	31.3	1	0.97
	Yes	9	33.3	1.10 (0.23–5.31)	
	Unknown	65	33.9	1.13 (0.45–2.79)	
Severe tiredness	No	77	35.1	1	0.69
	Yes	26	30.8	0.82 (0.32–2.14)	
Fever	No	92	30.4	1	0.033
	Yes	11	63.6	4.00 (1.08–14.76)	
Night-sweating	No	77	35.1	1	0.69
	Yes	26	30.8	0.82 (0.32–2.14)	
Diarrhea	No	100	34.0	1	0.98
	Yes	3	33.3	0.97 (0.09–11.09)	
Having a steady partner that inject drugs	No steady partner	66	24.2	1	0.033
	Steady partner who injected drugs now or ever	14	42.9	2.34 (0.71–7.77)	
	Steady partner who never injected drugs	25	52.0	3.39 (1.28–8.89)	
Injecting drug use in the previous 6 months	No	13	38.5	1	0.66
	Yes	93	32.3	0.76 (0.23–2.53)	
Continuation of injecting drug use after HCV seroconversion (i.e., injecting drug use at first and second anti-HCV positive visit)	No	32	34.4	1	0.77
	Yes	73	31.5	0.88 (0.36–2.12)	
Alcohol use (any consumption in the previous 6 months)	No	18	38.6	1	0.60
	Yes	88	29.0	0.74 (0.24–2.28)	

#### Clinical symptoms

Of all HCV cases, 36.8% reported at least one of the clinical symptoms (jaundice, severe tiredness, fever, night-sweating, diarrhea) in the 4–6 months preceding the first anti-HCV positive visit, but except for fever, none of the examined symptoms were significantly associated with HCV viral clearance in univariate analysis (table 1). DU who reported fever were more likely to clear HCV than those who did not (OR 4.00, 95% CI 1.08–14.76). This association was borderline significant after adjustment for sex (adjusted OR (aOR) 3.80, 95% CI 0.99–14.61). To further evaluate the association between viral clearance and clinical symptoms that might have occurred around the time of HCV seroconversion, we determined the incidence rate ratios of clinical symptoms on different visits shortly before and following HCV seroconversion (see methods section). In line with logistic regression analysis, the incidence rate of each of symptom did not significantly differ between those individuals who spontaneously cleared HCV and those individuals who developed chronic infection, except for fever (data not shown).

#### Co-infections

At the time of HCV seroconversion (or ACS entry in those already HCV-positive), 13 DU were HIV-co-infected. In univariate analysis, spontaneous HCV clearance was more likely in HIV-negative individuals than in HIV-positive individuals (OR 3.03, 95% CI 0.63–14.51), although the difference did not reach statistical significance, P = 0.13 (table 2). The effect of HIV co-infection did not change after adjusting for sex (aOR 3.48, 95% CI 0.70–17.40). CD4 and CD8 T-cell counts were available for 51 of the 106 HCV seroconverters and only 1 DU had a CD4 count below 350 cells/mL. The median CD4 and CD8 count did not differ between participants who cleared HCV and those who developed chronic HCV infection 935 (IQR 710–1,180) and 990 (IQR 770–1,180) CD4^+^ cells/mL, and 60 (IQR 45–85) and 70 (IQR 40–70) CD8^+^ cells/mL, respectively). For those with detectable HCV viral load at the first available sample after ACS entry or HCV seroconversion and who developed persistent viremia, HCV viral load tended to be lower in women than in men, but this effect was not statistically significant (median log 3.43 copies/mL (IQR 3.00–4.70) and 4.36 copies/mL (IQR 3.00–5.43), respectively (P = 0.14).

**Table 2:** Univariate analysis of host genetic factors and viral coinfections associated with HCV clearance in a cohort of 106 individuals with acute HCV acquired through injection drug use.

		N	Clearance rate (%)	OR (95%CI)	P value
HCV genotype	1	34	11.9	1	0.34
	2	6	0.0	-	
	3	28	14.3	1.25 (0.28–5.53)	
	4	6	16.7	1.50 (0.14–16.32)	
	Untypable (due to low viral load)	6	50.0	7.15 (1.11–50.66)	
Log HCV viral load	≤3	19	26.3	8.93 (0.95–84.30)	0.12
	3–4.9	26	3.9	1	
	≥4.9	27	11.1	3.13 (0.30–32.20)	
HIV-1	Presence of HIV-1 antibodies	13	15.4	1	0.13
	Absence of HIV-1 antibodies	93	35.5	3.03 (0.63–14.47)	
HBV co-infection	Anti-HBc-negative, HBsAg-negative	69	33.3	1	0.025
	Anti-HBc-positive, HBsAg-negative	27	19.2	0.48 (0.16–1.43)	
	Anti-HBc-positive, HBsAg-positive	8	75.0	6.00 (1.12–32.09)	
rs8099917	GG/TG	30	20.0	1	0.084
	TT	70	37.1	2.36 (0.85–6.54)	
rs12979860	TT/CT	48	22.9	1	0.060
	CC	52	40.4	3.55 (0.95–5.45)	

HBV, hepatits B virus; HCV, hepatitis C virus; HBc, hepatitis B core; HBsAG, Hepatitis B surface Antigen; IL28B, Interleukin 28B.

Of all patients with acute HCV, 27 had evidence of cleared HBV infection, (i.e., they were anti-HBc-positive and HBsAg-negative), and 8 had a chronic HBV infection (i.e., were HBsAg-positive and anti-HBc-positive). In univariate analysis, those with chronic HBV infection were more likely to clear HCV spontaneously (OR 6.00, 95% CI 1.12–32.1) than those never exposed (P = 0.04). After adjusting for sex, those with chronic HBV infection were still more likely to clear HCV spontaneously, although this effect was borderline significant. (aOR 5.00, 95% CI 0.88–28.36), P = 0.07.

#### Il28 B genotypes and spontaneous viral clearance

**Figure 1:**
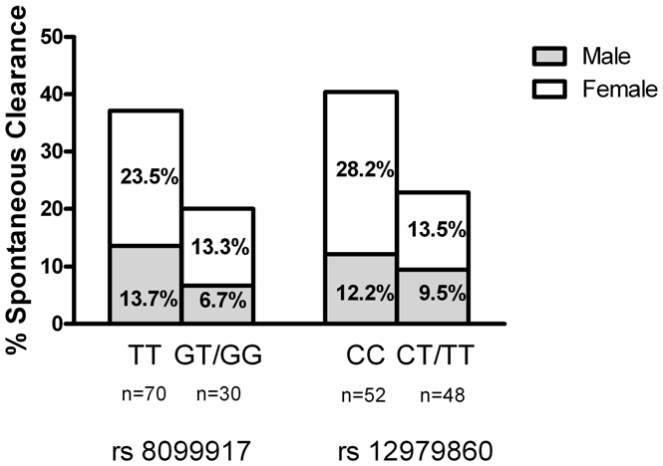
Distribution of sex for each genotype plotted by spontaneous HCV clearance rate. Genotyping was available for 100/106 participants. Bars represent the total percentage of spontaneous HCV clearance for the protective alleles (TT for rs8099917 and CC for rs12979860) and non-protective alleles (CT/GG for rs8099917 and CT/TT for rs12979860). The numbers in the bars indicate the percentage of spontaneous HCV clearance for males and females for each allele.

Data on both SNPs, rs8099917 and rs12979860, was available for 100/106 participants. Allele frequencies of both SNPs were comparable to those reported elsewhere in Europe [19], [22], rs8099917 (T = 0.84, G = 0.16) and rs12979860 (C = 0.70, T = 0.30). The CC genotype frequency of rs12979860 were comparable between males (CC = 0.55) and females (CC = 0.49). For rs8099917 the TT genotype frequency was also comparable for males (TT = 0.66) and females (TT = 0.74). Participants with the TT genotype of rs8099917 and the CC genotype of rs12979860 were more likely to have cleared the virus than those with the GG/TG and TT/CT genotype respectively (rs8099917 OR 2.36, 95% CI 0.85–6.54, rs12979860 OR 3.55, 95% CI 0.95–5.45), although the effects were borderline significant (table 2). Since female sex is a strong predictor for spontaneous clearance, we examined the effect of sex on clearance separately for each Il28B genotype and their alleles (figure 1). Women were 2.3 times more likely to clear HCV than men with the favorable genotype for rs12979860 (female/male clearance ratio 2.31 (28.2%/12.2%)). Whereas this ratio for the unfavorable TT/CT genotype was 1.39 (13.5/9.7), suggesting an interaction between sex and rs12979860. This interaction was not found for rs8099917 (TT 1.72 (23.5%/13.7%), GT/GG 1.89 (13.3%/6.7%)).

Next, we investigated the interaction between sex and each SNP in a logistic regression model. We found a potential interaction between rs12979860 and sex. Males with the favorable CC genotype had a somewhat increased odds to spontaneous clear HCV (OR 1.58, 95% CI 0.46–5.44) as compared to the reference group (males without the favorable CC genotype), although this effect was not significant. Females without the favorable CC genotype were as likely as men with the favorable genotype to spontaneously clear HCV (OR 1.58, 95% CI 0.43–6.10). Interestingly, females with the favorable CC genotype had an increased odds to spontaneous clear HCV of OR 7.20 (95% CI 1.87–27.75) as compared to the reference group, overall P = 0.015.

#### Multivariate analysis

Because of the interaction of rs12979860 and sex we used the combined variable in our final multivariate analyses. Females with CC genotype for rs12979860 had increased odds to spontaneously clear HCV infection (aOR 6.62, 95% CI 2.69–26.13) when compared to males without the favorable genotype (table 3). Males with the favorable genotype and females without the unfavorable genotype had comparable risks as men with the unfavorable genotype to clear the virus. Those who reported fever in the period preceding the first anti-HCV positive visit were also more likely to spontaneously clear HCV infection (OR 5.03, 95% CI 1.24–20.33). Further adjustment for HIV and HBV infection did not substantially change these results. Absence of HIV and presence of a chronic HBV infection were still borderline associated with viral clearance in this multivariate model, resp. (aOR 6.32, 95% CI 0.86–46.25) and (aOR 8.72, 95% CI 0.99–76.37).

**Table 3:** Multivariate analysis of factors associated with HCV clearance in a cohort of 106 individuals with acute HCV acquired through injection drug use.

		OR	95% CI	P value
Sex*rs12979860	Male, TT/CT	1		0.021
	Male, CC	1.34	0.37–4.85	
	Female, TT/CT	1.14	0.28–4.76	
	Female, CC	6.62	2.69–26.13	
Fever	No	1		0.023
	Yes	5.03	1.24–20.33	

In a sensitivity analysis including only HCV seroconverters with a seroconversion interval ≤6 months (n = 36), the results for the interaction rs12979860/sex and fever were comparable to the results from the multivariate model. The rate of spontaneous clearance was 36.1% (95% CI 20.3–52.3%). In an additional analysis including only West-Europeans (n = 86), females with the favourable CC genotype for rs12979860 had an increased odd to spontaneously clear their HCV infection (aOR 5.17, 95% CI 1.21–22.13) as compared to males without the favourable genotype.

## Discussion

In this study, the clearance rate and factors influencing spontaneous HCV clearance were assessed in a prospective cohort of retrospectively identified DU with acute HCV, regardless of their clinical presentation at the time of acute HCV infection. To our knowledge, this is one the largest longitudinal studies on factors associated with spontaneous HCV clearance in individuals with drug-use-related acute HCV infection. The rate of spontaneous HCV clearance was 33.0%. Our main finding is that women with the favorable genotype for rs12989760 were more likely to clear HCV (OR 6.62, 95% 2.69–26.13), whereas females with the unfavorable genotype were as likely as men with the favorable and unfavorable genotype to clear HCV.

The rate of clearance we found is higher than observed in studies among acute clinical cases (reviewed by Micallef [30]), but may be underestimated in injecting DU. Many injecting DU experience repeated exposure to HCV and HCV re-infection after their initial HCV seroconversion, due to continuing risk behavior [28]. Such re-infection after clearance might result in persistent or recurrent HCV viremia, leading to an underestimation of the clearance rate. However, in this cohort of DU with acute HCV, we did not find an association between ongoing risk behavior and reduced rates of viral clearance shortly after the initial HCV infection.

Studies have suggested that individuals presenting with clinical symptoms after exposure to HCV after needle-stick injury or presenting at an outpatient clinic are more likely to spontaneously resolve acute HCV infection [7], [31]. Self-reported fever was associated with HCV viral clearance in this cohort of DU, other clinical symptoms were not.

The association between HCV clearance and absence of HIV was borderline. HIV infection has been associated with loss of viral control of HCV, as evidenced by a higher HCV viral load in HIV co-infected individuals [32]. Since HCV is more efficiently transmitted by an infected needle stick than HIV, HCV usually precedes or coincides with HIV infection in DU. Therefore, the number of participants with HIV at HCV seroconversion was small, limiting the power to detect an effect. In addition, therefore all HIV co-infected DU in our study retained high CD4 counts at the point of HCV infection. This might explain why HCV viral load did not significantly differ between co-infected and mono-infected individuals in our study. As in line with our findings that early HIV infection already lowers the HCV clearance rate. We and others have shown that acute HIV co-infection hampers the beneficial HCV-specific CD4^+^ T-cell responses targeting non-structural proteins in DU [33]–[37].

Interestingly, chronic HBV was borderline significantly associated with HCV clearance in the multivariate model, while cases were limited. Patients with spontaneous viral clearance of chronic HCV after HBV-superinfection have been described [38], and cross-sectional studies have shown that HBsAg-positive HIV-infected HCV-seropositive individuals are more likely to be HCV-RNA-negative than HBsAg-negative HIV-infected individuals [39]–[41]. Although the effect on liver disease is unknown, chronic HBV infection seems to favor clearance of acute HCV infection [42]. Further studies on viral interference including the role of HBV, which may modify HCV replication are warranted.

Having defined viral clearance as two consecutive HCV-RNA-negative visits after anti-HCV seroconversion, we defined HCV viral persistence as the continuous presence of HCV RNA at one or two of these visits, regardless of HCV strain present. Therefore, we did not distinguish between viral persistence of one strain and reinfection by another. Furthermore, although HCV clearance is believed to take place within the first 6 months after acute infection, evidence shows that clearance might take much longer [10], [43]. Since we included HCV RNA measurements only in the first 2 years after HCV seroconversion, we recognize that multiple measurements in a longer time span would be necessary to evaluate possible late clearance and its predictors. In our cohort, after a median follow-up after HCV seroconversion of 14.6 years (IQR 7.9–19.6), only 5 out of 71 (7.0%) individuals who did not clear HCV spontaneously within the first 2 years after HCV seroconversion were HCV RNA-negative at the last study visit, without HCV treatment in the meantime, before November 2005 or the penultimate visit preceding death, indicating that late clearance might occur, but is not very frequent (data not shown).

Our main finding is the potential interaction between the favourable genotype of rs12989760 and women in spontaneous clearance of HCV. However, since our study population is relatively small, this finding needs to be confirmed in larger studies among HCV seroconverters. A possible interaction between IL28B and sex in HCV fibrosis progression has been described previously by Falletti et al. [44] This retrospective study among 629 cHCV infected patients investigated the role of IL28B on the histological outcome of cHCV infection. One of their findings is that males carrying the favourable C-allele for rs12979860 had a significant increased risk (OR 2.06) for an Ishak staging score >2 as compared to females with the favourable C-allele (reference category), while participants carrying the TT genotype also had an increased risk (OR 2.37) for an Ishak score>2, irrespective of sex.

The potential interaction between female gender and Il28B might be explained by the involvement of toll like receptor 7 (TLR7), a receptor that is involved in recognition of viral products (single stranded RNA) and activation of innate immunity [45]. Stimulation of TLR7 in peripheral blood mononuclear cells (PBMC) from women results in significantly higher IFN-α responses as compared to males [46]. The TLR7 activation signal is transduced via MyD88 to the IL-1R-associated kinase 1/4 complex that activates interferon regulatory factor 7 (IRF7). Recently, it has been demonstrated that IL28B (IFN-λ) is mainly controlled by IRF7 [47]. Interestingly, IFN-λ mediates its antiviral activity through the activation of JAK-STAT pathways, similar to IFN-α which is still the major treatment modality for cHCV infection, thereby inducing interferon stimulating genes (ISG) that suppress viral activity. Marcello et al showed, in vitro, that co treatment with both IFN-α and IFN-λ enhanced the antiviral activity, suggestive of a synergistic interaction [48]. Whether increased reactivity upon TLR7 stimulation results in both increased IFN-α and IFN-λ responses needs to be investigated.

Next to the relatively small sample size, our study is limited by the fact that variables on behaviour and symptoms are self-reported, including initiation of injecting drug use for those that entered our cohort as anti-HCV positive cases. We believed that we could minimize this bias by only including IDU who started injecting within two years before inclusion into the study. In an earlier report, we have shown that approximately 50% of IDU in the ACS become infected with HCV within two years after initiating injecting drug use and self-reports are valid [26], [49]. In addition, in a sensitivity analysis including only HCV seroconverters with a small interval between last anti-HCV negative and first anti-HCV positive test, results were comparable.

In conclusion, women with the favorable CC genotype for rs12979860 have the greatest likelihood to spontaneously resolve HCV. The decision to start HCV treatment might be postponed in this group, if not coinfected with HIV. Spontaneous clearance of HCV seems to be primarily driven by host genetic factors and presence of coinfection. The possible synergistic interaction between female sex and the favorable genotype warrants confirmation in larger studies and warrants further study into the immunological and virological mechanisms explaining this finding.
